# Chlorido(η^6^-*N* ^2^-diphenylphosphanyl-*N* ^1^,*N* ^1^-diisopropyl-4-methoxybenz­amidine-κ*P*)(triphenylphosphane-κ*P*)ruthenium(II) trifluoromethansulfonate acetone disolvate

**DOI:** 10.1107/S1600536813029450

**Published:** 2013-11-13

**Authors:** Manel Kéchaou-Perrot, Laure Vendier, Alain Igau

**Affiliations:** a Laboratoire de Chimie de Coordination, UPR-CNRS 8241, 205, route de Narbonne, 31077 Toulouse cedex, France

## Abstract

In the title compound, [RuCl(C_18_H_15_P)(C_26_H_31_N_2_OP)](CF_3_O_3_S)·2C_3_H_6_O, the Ru^II^ ion is coordinated in a three-legged piano stool, half-sandwich-type geometry by a chlorido ligand, a tri­phenyl­phosphine and a tethered η^6^-(phenyl-*p*-*O*-meth­oxy) κ^1^-*P N*-di­phenyl­phosphino *N*′-diisopropyl amidine ligand charge-balanced by a trifluormethansulfonate counter-anion. The η^6^-coordination mode of the arene incorporated into the structure was generated *in situ* after addition of methyl tri­fluoro­methane­sulfonate to the neutral η^5^-arene tethered precursor complex [RuCl(PPh_3_)(η^5^:κ^1^-OC_6_H_4_C(NiPr_2_)=N-PPh_2_)] in di­chloro­methane solution.

## Related literature

For related tethered η^6^-arene ruthenium(II) half-sandwich piano-stool complexes, see: Therrien & Ward (1999 ▶); Faller & D’Alliessi (2003 ▶); Cetinkaya *et al.* (2003 ▶); Cadierno *et al.* (2004 ▶); Ito *et al.* (2008 ▶); Arquier *et al.* (2009 ▶); Parekh *et al.* (2012 ▶). For η^5^-arene ruthenium(II) half-sandwich piano-stool complexes, see: Cole-Hamilton *et al.* (1976 ▶); Rosete *et al.* (1979 ▶); Snelgrove *et al.* (2005 ▶); Ferrando-Miguel *et al.* (2005 ▶). For the increasing medicinal inter­est in η^6^-arene ruth­enium(II) half-sandwich complexes, see: Hartinger & Dyson (2009 ▶); Allardyce *et al.* (2001 ▶); Scolaro *et al.* (2005 ▶); Dyson (2007 ▶); Chatterjee *et al.* (2008 ▶). For the synthesis of the precursor, see: Kechaou *et al.* (2013 ▶). 
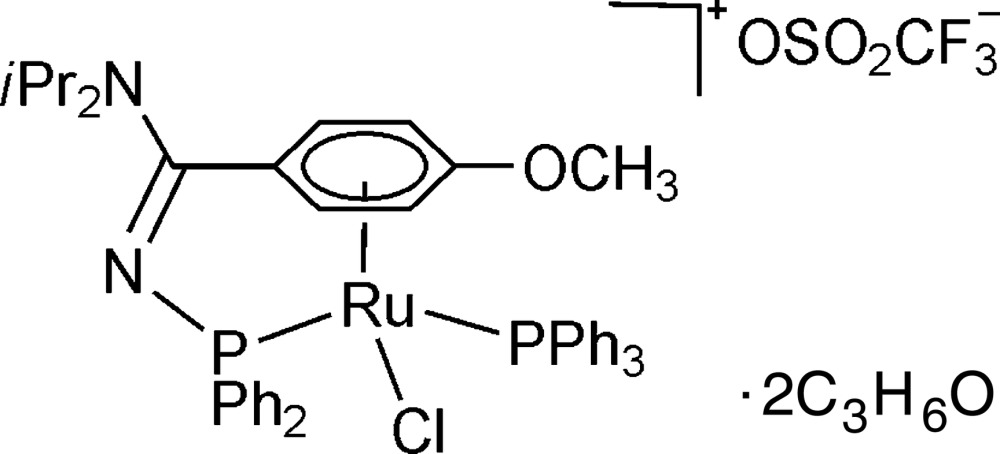


## Experimental

### 

#### Crystal data

[RuCl(C_18_H_15_P)(C_26_H_31_N_2_OP)](CF_3_O_3_S)·2C_3_H_6_O
*M* *_r_* = 1082.51
Monoclinic, 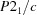

*a* = 11.6970 (2) Å
*b* = 15.0260 (3) Å
*c* = 29.7770 (6) Å
β = 99.864 (2)°
*V* = 5156.21 (17) Å^3^
*Z* = 4
Mo *K*α radiation
μ = 0.52 mm^−1^
*T* = 180 K
0.19 × 0.1 × 0.03 mm

#### Data collection

Oxford Diffraction Xcalibur (Eos, Gemini ultra) diffractometer
Absorption correction: multi-scan (*CrysAlis RED*; Oxford Diffraction, 2010 ▶) *T*_min_ = 0.933, *T*_max_ = 0.982
43527 measured reflections
10532 independent reflections
8192 reflections with *I* > 2σ(*I*)
*R*_int_ = 0.046

#### Refinement

*R*[*F* ^2^ > 2σ(*F* ^2^)] = 0.043
*wR*(*F* ^2^) = 0.089
*S* = 1.09
10532 reflections
608 parameters
H-atom parameters constrained
Δρ_max_ = 0.49 e Å^−3^
Δρ_min_ = −0.37 e Å^−3^

### 

Data collection: *CrysAlis CCD* (Oxford Diffraction, 2010 ▶); cell refinement: *CrysAlis CCD*; data reduction: *CrysAlis RED* (Oxford Diffraction, 2010 ▶); program(s) used to solve structure: *SIR92* (Altomare *et al.*, 1994 ▶); program(s) used to refine structure: *SHELXL97* (Sheldrick, 2008 ▶); molecular graphics: *ORTEP-3 for Windows* (Farrugia, 2012 ▶); software used to prepare material for publication: *WinGX* publication routines (Farrugia, 2012 ▶).

## Supplementary Material

Crystal structure: contains datablock(s) global, I. DOI: 10.1107/S1600536813029450/cq2007sup1.cif

Structure factors: contains datablock(s) I. DOI: 10.1107/S1600536813029450/cq2007Isup2.hkl

Supplementary material file. DOI: 10.1107/S1600536813029450/cq2007Isup3.cdx

968713

Additional supplementary materials:  crystallographic information; 3D view; checkCIF report

## Figures and Tables

**Fig. 1.**
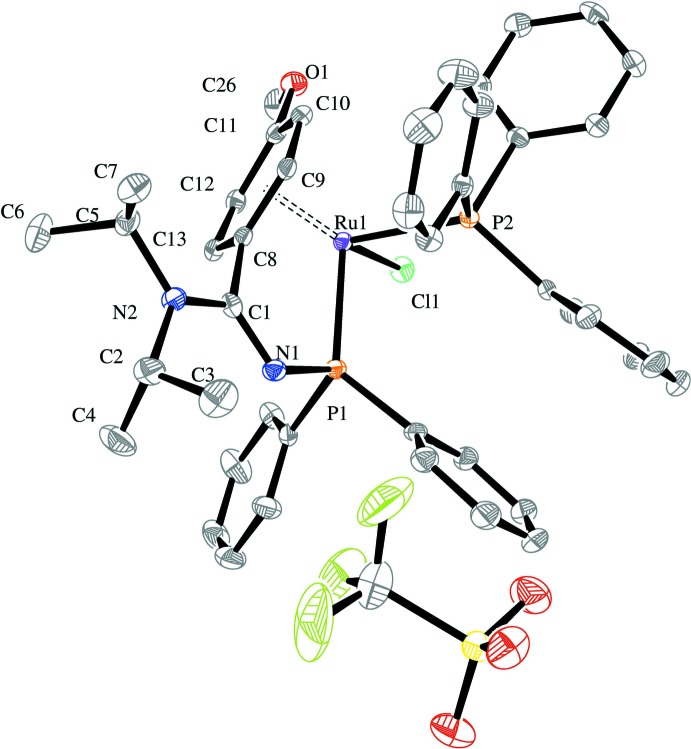
A molecule of the title complex with atom labelling scheme and 30% probability displacement ellipsoids.

## supplementary crystallographic information

### 1. Comment

In our efforts to prepare piano stool, half-sandwich, tethered functionalized η^6^-arene ruthenium(II) complexes, we investigated the reaction of the ruthenium(II) precursor complex RuCl(PPh3)(η^5^:κ^1^-OC_6_H_4_C(NiPr_2_)=N-PPh_2_)] with methyl trifluoromethanesulfonate. The aim was to prepare quantitatively, as assessed by NMR spectroscopy, the corresponding functionalized η^6^-arene methoxy ruthenium(II) complex. It is noteworthy that alkylation on the carbonyl function of a coordinated η^5^-oxocyclohexadienyl ligand had never been reported before our studies. The title complex was isolated as a dark-yellow powder and crystals of suitable quality for XRD analysis were obtained by slow diffusion of pentane into an acetone solution of the complex. Single crystal X-ray analysis determined the structure of the new three-legged piano stool half-sandwich type complex. The ruthenium(II) ion is coordinated to a chloro ligand, a triphenylphosphine and a tethered η^6^-(phenyl-*p*-*O*-methoxy) κ^1^-P *N*-diphenylphosphino *N*'-diisopropyl amidine ligand. The ruthenium adopts a pseudo-tetrahedral geometry with the P1—Ru1—Cl1, P1—Ru1—Cl2, and Cl2—Ru1—Cl1 bond angles close to 90 °. As expected, the carbon atoms of the η^6^-coordinated ring are coplanar. It is proposed that there are π-π intramolecular arene interactions between the phenyl ring (C14, C15, C16, C17, C18, C19) coordinated to P1 and the phenyl ring (C39, C40, C41, C42, C43, C44) on P2. The distance beween the centroids of the two rings is 3.609 Å, their average interplane distance is 3.397 Å and their offset angle α is 19.9 °. The title complex was fully characterized by infrared spectroscopy, ^1^H, ^13^C and ^31^P NMR spectroscopy, together with mass spectrometry. The formation of the title compound opens up a synthetic route for the preparation of a large variety of tethered three-legged piano stool, half-sandwich functionalized η^6^-arene ruthenium(II) complexes, which will be tested both as catalysts and anticancer drugs. Currently, η^6^-arene ruthenium-based anticancer chemotherapies are making significant advances in clinical trials.

### 2. Experimental

All manipulations were carried out in dry solvents and under dry argon atmosphere. The precursor complex [RuCl(PPh3)(η^5^:κ^1^-OC_6_H_4_C(NiPr_2_)=N-PPh_2_)] was prepared according to the previously described experimental procedure of Kechaou *et al.*(2013). Methyl trifluoromethanesulfonate was purchased from Aldrich and used as received without further purification. Methyl trifluoromethanesulfonate (0.025 ml; 0.230 mmol; 1 eq) was added at room temperature on the precursor complex [RuCl(PPh_3_)(η^5^:κ^1^-OC_6_H_4_C(NiPr_2_)=N-PPh_2_)] (0.185 g; 0.230 mmol) dissolved in dichloromethane (15 ml) using standard Schlenk-line and cannula techniques under dry argon atmosphere. The reaction mixture was stirred for 2 h at room temperature. After removal of the volatiles, the residue was washed with 2 *x* 15 ml of ether and dried under vacuum to give a dark-yellow powder of the title compound [RuCl(PPh_3_)(η^6^:κ^1^-MeOC_6_H_4_C(NiPr_2_)=N-PPh_2_)] (OSO_2_CF_3_)(0.200 mg, 90% isolated yield). Dissolution of 30 mg of the resulting dark yellow powder in 0.5 ml of dry acetone followed by careful diffusion of pentane as a non solvent into the resulting solution afforded at room temperature, crystals of the title compound suitable for XRD analysis.

### 3. Refinement

The H atoms were positioned geometrically (C—H = 0.95 - 1.00 Å) and refined as riding on their parent atoms, with U(H) = 1.2 *x U*_eq_(carrier).

### Crystal data

**Table:**

[RuCl(C_18_H_15_P)(C_26_H_31_N_2_OP)](CF_3_O_3_S)·2C_3_H_6_O	*F*(000) = 2240
*M**_r_* = 1082.51	*D*_x_ = 1.394 Mg m^−^^3^
Monoclinic, *P*2_1_/*c*	Mo *K*α radiation, λ = 0.71073 Å
Hall symbol: -P 2ybc	Cell parameters from 14433 reflections
*a* = 11.6970 (2) Å	θ = 3.3–29.1°
*b* = 15.0260 (3) Å	µ = 0.52 mm^−^^1^
*c* = 29.7770 (6) Å	*T* = 180 K
β = 99.864 (2)°	Parallelepiped, yellow
*V* = 5156.21 (17) Å^3^	0.19 × 0.1 × 0.03 mm
*Z* = 4	

### Data collection

**Table:**

Oxford Diffraction Xcalibur (Eos, Gemini ultra) diffractometer	10532 independent reflections
Graphite monochromator	8192 reflections with *I* > 2σ(*I*)
Detector resolution: 16.1978 pixels mm^-1^	*R*_int_ = 0.046
ω scans	θ_max_ = 26.4°, θ_min_ = 3.3°
Absorption correction: multi-scan (*CrysAlis RED*; Oxford Diffraction, 2010)	*h* = −14→14
*T*_min_ = 0.933, *T*_max_ = 0.982	*k* = −18→18
43527 measured reflections	*l* = −37→36

### Refinement

**Table:**

Refinement on *F*^2^	Primary atom site location: structure-invariant direct methods
Least-squares matrix: full	Secondary atom site location: difference Fourier map
*R*[*F*^2^ > 2σ(*F*^2^)] = 0.043	Hydrogen site location: inferred from neighbouring sites
*wR*(*F*^2^) = 0.089	H-atom parameters constrained
*S* = 1.09	*w* = 1/[σ^2^(*F*_o_^2^) + (0.0251*P*)^2^ + 6.3129*P*] where *P* = (*F*_o_^2^ + 2*F*_c_^2^)/3
10532 reflections	(Δ/σ)_max_ = 0.001
608 parameters	Δρ_max_ = 0.49 e Å^−^^3^
0 restraints	Δρ_min_ = −0.37 e Å^−^^3^

### Special details

**Table:**

Geometry. All e.s.d.'s (except the e.s.d. in the dihedral angle between two l.s. planes) are estimated using the full covariance matrix. The cell e.s.d.'s are taken into account individually in the estimation of e.s.d.'s in distances, angles and torsion angles; correlations between e.s.d.'s in cell parameters are only used when they are defined by crystal symmetry. An approximate (isotropic) treatment of cell e.s.d.'s is used for estimating e.s.d.'s involving l.s. planes.
Refinement. Refinement of *F*^2^ against ALL reflections. The weighted *R*-factor *wR* and goodness of fit *S* are based on *F*^2^, conventional *R*-factors *R* are based on *F*, with *F* set to zero for negative *F*^2^. The threshold expression of *F*^2^ > σ(*F*^2^) is used only for calculating *R*-factors(gt) *etc*. and is not relevant to the choice of reflections for refinement. *R*-factors based on *F*^2^ are statistically about twice as large as those based on *F*, and *R*- factors based on ALL data will be even larger.

### Fractional atomic coordinates and isotropic or equivalent isotropic displacement parameters (Å^2^)

**Table:**

	*x*	*y*	*z*	*U*_iso_*/*U*_eq_	
C1	0.7448 (2)	0.64736 (19)	0.33547 (10)	0.0191 (6)	
C2	0.9016 (3)	0.5770 (2)	0.38976 (11)	0.0304 (7)	
H2	0.94	0.5174	0.3911	0.036*	
C3	0.8536 (3)	0.5865 (3)	0.43390 (11)	0.0438 (9)	
H3A	0.7945	0.5407	0.4351	0.066*	
H3B	0.8187	0.6456	0.4351	0.066*	
H3C	0.9167	0.5793	0.4599	0.066*	
C4	0.9954 (3)	0.6449 (3)	0.38473 (14)	0.0451 (10)	
H4A	1.0228	0.6349	0.3558	0.068*	
H4B	1.0604	0.6383	0.4101	0.068*	
H4C	0.9633	0.7051	0.3851	0.068*	
C5	0.7945 (3)	0.4909 (2)	0.32253 (10)	0.0258 (7)	
H5	0.7267	0.4988	0.2974	0.031*	
C6	0.9004 (3)	0.4730 (2)	0.30033 (13)	0.0407 (9)	
H6A	0.9139	0.5241	0.2814	0.061*	
H6B	0.8869	0.4196	0.2812	0.061*	
H6C	0.9686	0.4638	0.324	0.061*	
C7	0.7657 (3)	0.4149 (2)	0.35213 (12)	0.0344 (8)	
H7A	0.6966	0.4301	0.3651	0.052*	
H7B	0.8312	0.4045	0.3769	0.052*	
H7C	0.7506	0.3609	0.3336	0.052*	
C8	0.6649 (2)	0.64521 (18)	0.28973 (9)	0.0175 (6)	
C9	0.5538 (2)	0.60572 (18)	0.28247 (10)	0.0185 (6)	
H9	0.5249	0.575	0.3082	0.022*	
C10	0.4784 (3)	0.61740 (18)	0.23983 (10)	0.0205 (6)	
H10	0.395	0.6004	0.2374	0.025*	
C11	0.5109 (3)	0.66996 (19)	0.20556 (9)	0.0204 (6)	
C12	0.6224 (2)	0.71251 (19)	0.21369 (10)	0.0201 (6)	
H12	0.6403	0.76	0.1924	0.024*	
C13	0.7009 (2)	0.69499 (18)	0.25352 (9)	0.0192 (6)	
H13	0.7749	0.7296	0.2599	0.023*	
C14	0.6426 (2)	0.86642 (18)	0.38598 (10)	0.0191 (6)	
C15	0.6010 (3)	0.95355 (19)	0.38091 (11)	0.0242 (7)	
H15	0.5835	0.9793	0.3514	0.029*	
C16	0.5853 (3)	1.0024 (2)	0.41863 (12)	0.0326 (8)	
H16	0.5574	1.0618	0.4151	0.039*	
C17	0.6101 (3)	0.9647 (2)	0.46174 (12)	0.0354 (8)	
H17	0.5991	0.9986	0.4876	0.042*	
C18	0.6506 (3)	0.8787 (2)	0.46733 (11)	0.0345 (8)	
H18	0.6674	0.8532	0.4969	0.041*	
C19	0.6669 (3)	0.8291 (2)	0.42919 (10)	0.0269 (7)	
H19	0.6947	0.7697	0.4329	0.032*	
C20	0.7844 (2)	0.88336 (18)	0.32068 (10)	0.0205 (6)	
C21	0.7796 (3)	0.9194 (2)	0.27773 (11)	0.0277 (7)	
H21	0.7153	0.9072	0.2544	0.033*	
C22	0.8695 (3)	0.9736 (2)	0.26888 (13)	0.0389 (9)	
H22	0.8669	0.9983	0.2393	0.047*	
C23	0.9623 (3)	0.9917 (2)	0.30272 (13)	0.0386 (9)	
H23	1.0229	1.0293	0.2964	0.046*	
C24	0.9680 (3)	0.9559 (2)	0.34557 (13)	0.0385 (9)	
H24	1.0325	0.9683	0.3687	0.046*	
C25	0.8796 (3)	0.9019 (2)	0.35462 (11)	0.0311 (7)	
H25	0.8834	0.877	0.3842	0.037*	
C26	0.4576 (3)	0.7429 (2)	0.13336 (11)	0.0361 (8)	
H26A	0.3943	0.7423	0.1071	0.054*	
H26B	0.5305	0.7271	0.1233	0.054*	
H26C	0.4644	0.8025	0.1469	0.054*	
C27	0.2384 (2)	0.75490 (18)	0.27688 (9)	0.0189 (6)	
C28	0.2233 (3)	0.73245 (18)	0.23098 (10)	0.0221 (6)	
H28	0.2892	0.7226	0.2169	0.027*	
C29	0.1127 (3)	0.72436 (19)	0.20550 (10)	0.0259 (7)	
H29	0.1033	0.7086	0.1742	0.031*	
C30	0.0166 (3)	0.7391 (2)	0.22557 (11)	0.0294 (7)	
H30	−0.0591	0.7333	0.2082	0.035*	
C31	0.0305 (3)	0.7624 (2)	0.27103 (11)	0.0291 (7)	
H31	−0.0358	0.7735	0.2847	0.035*	
C32	0.1399 (3)	0.76975 (19)	0.29674 (11)	0.0246 (7)	
H32	0.1485	0.785	0.3281	0.029*	
C33	0.3814 (2)	0.65650 (18)	0.34787 (10)	0.0192 (6)	
C34	0.4756 (3)	0.6391 (2)	0.38184 (10)	0.0239 (7)	
H34	0.5316	0.6842	0.3909	0.029*	
C35	0.4885 (3)	0.5561 (2)	0.40268 (11)	0.0327 (8)	
H35	0.5533	0.5445	0.4259	0.039*	
C36	0.4065 (3)	0.4902 (2)	0.38956 (12)	0.0391 (9)	
H36	0.4157	0.4332	0.4035	0.047*	
C37	0.3121 (3)	0.5075 (2)	0.35641 (13)	0.0408 (9)	
H37	0.2559	0.4623	0.3476	0.049*	
C38	0.2983 (3)	0.59039 (19)	0.33575 (11)	0.0271 (7)	
H38	0.232	0.6021	0.3133	0.033*	
C39	0.3551 (2)	0.84996 (18)	0.35094 (10)	0.0179 (6)	
C40	0.3472 (3)	0.8362 (2)	0.39642 (10)	0.0245 (7)	
H40	0.3655	0.7796	0.41	0.029*	
C41	0.3125 (3)	0.9056 (2)	0.42219 (11)	0.0323 (8)	
H41	0.3073	0.8958	0.4533	0.039*	
C42	0.2858 (3)	0.9876 (2)	0.40318 (12)	0.0317 (8)	
H42	0.262	1.0344	0.4209	0.038*	
C43	0.2936 (3)	1.0015 (2)	0.35794 (11)	0.0296 (7)	
H43	0.2751	1.0583	0.3447	0.035*	
C44	0.3281 (3)	0.93381 (19)	0.33178 (11)	0.0246 (7)	
H44	0.3334	0.9443	0.3007	0.03*	
C50	0.8526 (4)	0.6788 (3)	0.56855 (15)	0.0553 (11)	
C66	0.6369 (3)	0.3577 (3)	0.48420 (13)	0.0424 (9)	
C67	0.7339 (4)	0.3712 (3)	0.52314 (14)	0.0592 (12)	
H67A	0.7881	0.4154	0.5147	0.089*	
H67B	0.7747	0.3147	0.5306	0.089*	
H67C	0.7027	0.3924	0.5497	0.089*	
C68	0.5406 (4)	0.2982 (3)	0.49175 (13)	0.0580 (12)	
H68A	0.4793	0.2993	0.4648	0.087*	
H68B	0.5092	0.3186	0.5184	0.087*	
H68C	0.5698	0.2372	0.497	0.087*	
C69	0.8491 (5)	0.1454 (4)	0.4658 (2)	0.0786 (16)	
C70	0.8658 (6)	0.1162 (6)	0.4222 (3)	0.175 (5)	
H70A	0.8907	0.0538	0.424	0.263*	
H70B	0.9254	0.1528	0.4118	0.263*	
H70C	0.7928	0.1216	0.4007	0.263*	
C71	0.9401 (7)	0.1297 (7)	0.5068 (3)	0.190 (5)	
H71A	1.0076	0.1674	0.505	0.285*	
H71B	0.9636	0.0671	0.5078	0.285*	
H71C	0.9089	0.1444	0.5344	0.285*	
N1	0.7535 (2)	0.72111 (15)	0.35940 (8)	0.0198 (5)	
N2	0.8092 (2)	0.57553 (15)	0.34877 (8)	0.0213 (5)	
O1	0.43348 (18)	0.67901 (14)	0.16692 (7)	0.0263 (5)	
O2	0.7757 (2)	0.70212 (17)	0.64312 (8)	0.0464 (7)	
O3	0.8895 (2)	0.81958 (17)	0.61752 (9)	0.0490 (7)	
O4	0.6950 (2)	0.79177 (18)	0.57825 (9)	0.0502 (7)	
O5	0.7618 (4)	0.1818 (3)	0.47194 (14)	0.1011 (13)	
O6	0.6368 (3)	0.3922 (2)	0.44746 (10)	0.0660 (9)	
P1	0.67390 (6)	0.80800 (5)	0.33617 (2)	0.01691 (15)	
P2	0.38071 (6)	0.75812 (5)	0.31383 (2)	0.01565 (15)	
S1	0.79746 (7)	0.75662 (6)	0.60604 (3)	0.02905 (17)	
Cl1	0.47598 (6)	0.89077 (5)	0.24250 (3)	0.02359 (16)	
Ru1	0.541225 (18)	0.750672 (15)	0.276075 (7)	0.01445 (6)	
F1	0.9469 (3)	0.6396 (3)	0.58828 (13)	0.1238 (14)	
F2	0.8779 (3)	0.7198 (2)	0.53197 (10)	0.0985 (11)	
F3	0.7758 (3)	0.61686 (19)	0.55310 (11)	0.0963 (11)	

### Atomic displacement parameters (Å^2^)

**Table:**

	*U*^11^	*U*^22^	*U*^33^	*U*^12^	*U*^13^	*U*^23^
C1	0.0157 (15)	0.0222 (15)	0.0200 (15)	0.0012 (11)	0.0046 (12)	0.0035 (12)
C2	0.0301 (18)	0.0293 (17)	0.0266 (17)	0.0070 (14)	−0.0094 (14)	0.0016 (14)
C3	0.056 (2)	0.048 (2)	0.0244 (19)	0.0118 (19)	−0.0037 (17)	0.0037 (16)
C4	0.0236 (19)	0.050 (2)	0.055 (2)	−0.0009 (16)	−0.0121 (17)	0.0023 (19)
C5	0.0280 (17)	0.0241 (16)	0.0236 (16)	0.0078 (13)	−0.0003 (13)	−0.0030 (13)
C6	0.049 (2)	0.0341 (19)	0.044 (2)	0.0098 (17)	0.0218 (19)	−0.0033 (17)
C7	0.042 (2)	0.0266 (17)	0.0345 (19)	−0.0002 (15)	0.0060 (16)	−0.0032 (15)
C8	0.0199 (15)	0.0151 (13)	0.0169 (14)	0.0050 (11)	0.0018 (12)	−0.0028 (11)
C9	0.0196 (15)	0.0146 (13)	0.0218 (15)	0.0036 (11)	0.0054 (12)	−0.0022 (12)
C10	0.0202 (16)	0.0174 (14)	0.0235 (16)	0.0007 (12)	0.0030 (13)	−0.0045 (12)
C11	0.0236 (16)	0.0217 (15)	0.0154 (14)	0.0044 (12)	0.0021 (12)	−0.0049 (12)
C12	0.0217 (15)	0.0215 (14)	0.0190 (15)	0.0016 (12)	0.0084 (12)	−0.0006 (12)
C13	0.0191 (15)	0.0178 (14)	0.0215 (15)	0.0011 (11)	0.0061 (12)	−0.0032 (12)
C14	0.0185 (15)	0.0201 (14)	0.0183 (15)	−0.0054 (12)	0.0023 (12)	−0.0045 (12)
C15	0.0250 (16)	0.0230 (15)	0.0254 (16)	−0.0038 (13)	0.0067 (13)	−0.0032 (13)
C16	0.0284 (18)	0.0292 (17)	0.041 (2)	−0.0048 (14)	0.0094 (16)	−0.0125 (15)
C17	0.0316 (19)	0.046 (2)	0.0299 (19)	−0.0055 (16)	0.0086 (15)	−0.0211 (16)
C18	0.0312 (19)	0.052 (2)	0.0202 (17)	−0.0044 (16)	0.0037 (14)	−0.0052 (15)
C19	0.0269 (17)	0.0304 (17)	0.0231 (16)	−0.0016 (14)	0.0031 (13)	−0.0014 (13)
C20	0.0163 (15)	0.0194 (14)	0.0271 (16)	−0.0002 (11)	0.0076 (13)	−0.0045 (12)
C21	0.0203 (16)	0.0284 (16)	0.0342 (18)	0.0007 (13)	0.0039 (14)	0.0056 (14)
C22	0.0297 (19)	0.039 (2)	0.049 (2)	−0.0015 (16)	0.0086 (17)	0.0215 (17)
C23	0.0243 (18)	0.0335 (19)	0.061 (3)	−0.0082 (15)	0.0157 (18)	0.0018 (18)
C24	0.0279 (19)	0.046 (2)	0.042 (2)	−0.0157 (16)	0.0072 (16)	−0.0125 (17)
C25	0.0250 (17)	0.044 (2)	0.0243 (17)	−0.0107 (15)	0.0038 (14)	−0.0059 (15)
C26	0.0392 (19)	0.046 (2)	0.0219 (16)	0.0074 (17)	0.0023 (14)	0.0078 (16)
C27	0.0176 (13)	0.0141 (13)	0.0241 (14)	−0.0003 (12)	0.0012 (11)	0.0016 (13)
C28	0.0212 (15)	0.0218 (16)	0.0236 (15)	0.0021 (12)	0.0044 (12)	0.0017 (12)
C29	0.0294 (17)	0.0260 (16)	0.0206 (16)	−0.0004 (13)	−0.0006 (13)	0.0016 (12)
C30	0.0169 (15)	0.0321 (18)	0.0364 (18)	−0.0040 (14)	−0.0038 (13)	0.0035 (15)
C31	0.0193 (15)	0.0329 (18)	0.0359 (18)	−0.0004 (13)	0.0067 (13)	−0.0024 (15)
C32	0.0211 (15)	0.0268 (17)	0.0261 (16)	−0.0025 (12)	0.0051 (13)	−0.0033 (13)
C33	0.0211 (15)	0.0182 (14)	0.0200 (15)	0.0014 (12)	0.0081 (12)	0.0021 (12)
C34	0.0244 (16)	0.0253 (16)	0.0218 (16)	−0.0017 (13)	0.0037 (13)	0.0028 (13)
C35	0.035 (2)	0.0354 (19)	0.0270 (18)	0.0088 (15)	0.0036 (15)	0.0093 (15)
C36	0.054 (2)	0.0227 (17)	0.041 (2)	0.0006 (16)	0.0093 (18)	0.0113 (15)
C37	0.047 (2)	0.0257 (18)	0.048 (2)	−0.0117 (16)	0.0049 (19)	0.0059 (16)
C38	0.0298 (18)	0.0222 (16)	0.0285 (17)	−0.0042 (13)	0.0029 (14)	0.0022 (13)
C39	0.0131 (14)	0.0195 (14)	0.0219 (15)	−0.0013 (11)	0.0049 (12)	−0.0042 (12)
C40	0.0284 (17)	0.0225 (15)	0.0228 (16)	−0.0016 (13)	0.0053 (13)	−0.0031 (13)
C41	0.040 (2)	0.0370 (19)	0.0218 (17)	−0.0055 (16)	0.0101 (15)	−0.0102 (14)
C42	0.0297 (18)	0.0280 (17)	0.038 (2)	0.0010 (14)	0.0091 (15)	−0.0142 (15)
C43	0.0321 (18)	0.0192 (15)	0.037 (2)	0.0043 (13)	0.0053 (15)	−0.0043 (14)
C44	0.0258 (17)	0.0241 (16)	0.0246 (16)	−0.0010 (13)	0.0062 (13)	0.0027 (13)
C50	0.065 (3)	0.052 (3)	0.054 (3)	0.012 (2)	0.025 (2)	−0.001 (2)
C66	0.048 (2)	0.045 (2)	0.032 (2)	0.0074 (18)	0.0013 (17)	0.0012 (17)
C67	0.055 (3)	0.071 (3)	0.047 (3)	−0.010 (2)	−0.003 (2)	−0.006 (2)
C68	0.049 (3)	0.087 (3)	0.036 (2)	−0.015 (2)	−0.0002 (19)	0.000 (2)
C69	0.085 (4)	0.060 (3)	0.099 (5)	−0.006 (3)	0.041 (4)	0.006 (3)
C70	0.124 (6)	0.267 (11)	0.164 (7)	−0.105 (7)	0.107 (6)	−0.131 (7)
C71	0.118 (7)	0.263 (13)	0.188 (10)	0.034 (8)	0.023 (7)	0.105 (9)
N1	0.0192 (13)	0.0201 (12)	0.0185 (12)	0.0007 (10)	−0.0012 (10)	−0.0019 (10)
N2	0.0218 (13)	0.0195 (12)	0.0205 (13)	0.0044 (10)	−0.0024 (11)	−0.0015 (10)
O1	0.0259 (12)	0.0333 (12)	0.0179 (11)	0.0001 (9)	−0.0014 (9)	−0.0020 (9)
O2	0.0642 (18)	0.0409 (15)	0.0380 (14)	−0.0084 (13)	0.0201 (13)	0.0028 (12)
O3	0.0382 (15)	0.0519 (16)	0.0543 (17)	−0.0170 (13)	0.0010 (13)	0.0013 (14)
O4	0.0375 (15)	0.0547 (16)	0.0525 (17)	0.0067 (13)	−0.0086 (13)	0.0005 (14)
O5	0.114 (3)	0.095 (3)	0.098 (3)	0.025 (3)	0.031 (3)	−0.019 (2)
O6	0.081 (2)	0.064 (2)	0.0507 (19)	0.0075 (17)	0.0058 (17)	0.0228 (16)
P1	0.0171 (4)	0.0170 (3)	0.0162 (4)	−0.0007 (3)	0.0016 (3)	−0.0014 (3)
P2	0.0156 (3)	0.0150 (3)	0.0165 (3)	−0.0006 (3)	0.0029 (3)	−0.0002 (3)
S1	0.0282 (4)	0.0303 (4)	0.0283 (4)	−0.0015 (4)	0.0040 (3)	0.0004 (4)
Cl1	0.0252 (4)	0.0190 (3)	0.0264 (4)	0.0033 (3)	0.0040 (3)	0.0059 (3)
Ru1	0.01440 (11)	0.01440 (10)	0.01446 (11)	0.00007 (9)	0.00221 (8)	−0.00016 (10)
F1	0.113 (3)	0.133 (3)	0.129 (3)	0.091 (2)	0.028 (2)	−0.004 (2)
F2	0.139 (3)	0.111 (2)	0.0626 (18)	−0.002 (2)	0.065 (2)	−0.0061 (17)
F3	0.139 (3)	0.0638 (18)	0.097 (2)	−0.0247 (19)	0.050 (2)	−0.0467 (17)

### Geometric parameters (Å, º)

**Table:**

C1—N1	1.312 (4)	C26—H26C	0.98
C1—N2	1.337 (4)	C27—C28	1.389 (4)
C1—C8	1.514 (4)	C27—C32	1.401 (4)
C2—N2	1.486 (4)	C27—P2	1.832 (3)
C2—C3	1.522 (5)	C28—C29	1.388 (4)
C2—C4	1.523 (5)	C28—H28	0.95
C2—H2	1	C29—C30	1.379 (4)
C3—H3A	0.98	C29—H29	0.95
C3—H3B	0.98	C30—C31	1.380 (4)
C3—H3C	0.98	C30—H30	0.95
C4—H4A	0.98	C31—C32	1.378 (4)
C4—H4B	0.98	C31—H31	0.95
C4—H4C	0.98	C32—H32	0.95
C5—N2	1.487 (4)	C33—C34	1.387 (4)
C5—C7	1.516 (4)	C33—C38	1.394 (4)
C5—C6	1.524 (4)	C33—P2	1.832 (3)
C5—H5	1	C34—C35	1.389 (4)
C6—H6A	0.98	C34—H34	0.95
C6—H6B	0.98	C35—C36	1.387 (5)
C6—H6C	0.98	C35—H35	0.95
C7—H7A	0.98	C36—C37	1.374 (5)
C7—H7B	0.98	C36—H36	0.95
C7—H7C	0.98	C37—C38	1.387 (4)
C8—C9	1.411 (4)	C37—H37	0.95
C8—C13	1.434 (4)	C38—H38	0.95
C8—Ru1	2.137 (3)	C39—C40	1.388 (4)
C9—C10	1.427 (4)	C39—C44	1.397 (4)
C9—Ru1	2.189 (3)	C39—P2	1.824 (3)
C9—H9	1	C40—C41	1.395 (4)
C10—C11	1.394 (4)	C40—H40	0.95
C10—Ru1	2.333 (3)	C41—C42	1.370 (5)
C10—H10	1	C41—H41	0.95
C11—O1	1.344 (3)	C42—C43	1.382 (5)
C11—C12	1.435 (4)	C42—H42	0.95
C11—Ru1	2.398 (3)	C43—C44	1.382 (4)
C12—C13	1.395 (4)	C43—H43	0.95
C12—Ru1	2.299 (3)	C44—H44	0.95
C12—H12	1	C50—F1	1.299 (5)
C13—Ru1	2.252 (3)	C50—F3	1.320 (5)
C13—H13	1	C50—F2	1.328 (5)
C14—C19	1.388 (4)	C50—S1	1.810 (4)
C14—C15	1.396 (4)	C66—O6	1.210 (4)
C14—P1	1.814 (3)	C66—C68	1.486 (5)
C15—C16	1.380 (4)	C66—C67	1.490 (5)
C15—H15	0.95	C67—H67A	0.98
C16—C17	1.388 (5)	C67—H67B	0.98
C16—H16	0.95	C67—H67C	0.98
C17—C18	1.377 (5)	C68—H68A	0.98
C17—H17	0.95	C68—H68B	0.98
C18—C19	1.398 (4)	C68—H68C	0.98
C18—H18	0.95	C69—O5	1.199 (6)
C19—H19	0.95	C69—C70	1.416 (8)
C20—C21	1.381 (4)	C69—C71	1.493 (9)
C20—C25	1.398 (4)	C70—H70A	0.98
C20—P1	1.836 (3)	C70—H70B	0.98
C21—C22	1.391 (4)	C70—H70C	0.98
C21—H21	0.95	C71—H71A	0.98
C22—C23	1.375 (5)	C71—H71B	0.98
C22—H22	0.95	C71—H71C	0.98
C23—C24	1.376 (5)	N1—P1	1.682 (2)
C23—H23	0.95	O2—S1	1.432 (2)
C24—C25	1.377 (4)	O3—S1	1.430 (2)
C24—H24	0.95	O4—S1	1.435 (3)
C25—H25	0.95	P1—Ru1	2.3240 (8)
C26—O1	1.448 (4)	P2—Ru1	2.3505 (7)
C26—H26A	0.98	Cl1—Ru1	2.3984 (7)
C26—H26B	0.98		
			
N1—C1—N2	122.3 (3)	C31—C32—H32	119.8
N1—C1—C8	119.0 (2)	C27—C32—H32	119.8
N2—C1—C8	118.6 (2)	C34—C33—C38	119.1 (3)
N2—C2—C3	112.7 (3)	C34—C33—P2	119.2 (2)
N2—C2—C4	111.4 (3)	C38—C33—P2	120.9 (2)
C3—C2—C4	113.6 (3)	C33—C34—C35	120.5 (3)
N2—C2—H2	106.2	C33—C34—H34	119.8
C3—C2—H2	106.2	C35—C34—H34	119.8
C4—C2—H2	106.2	C36—C35—C34	119.8 (3)
C2—C3—H3A	109.5	C36—C35—H35	120.1
C2—C3—H3B	109.5	C34—C35—H35	120.1
H3A—C3—H3B	109.5	C37—C36—C35	120.0 (3)
C2—C3—H3C	109.5	C37—C36—H36	120
H3A—C3—H3C	109.5	C35—C36—H36	120
H3B—C3—H3C	109.5	C36—C37—C38	120.4 (3)
C2—C4—H4A	109.5	C36—C37—H37	119.8
C2—C4—H4B	109.5	C38—C37—H37	119.8
H4A—C4—H4B	109.5	C37—C38—C33	120.2 (3)
C2—C4—H4C	109.5	C37—C38—H38	119.9
H4A—C4—H4C	109.5	C33—C38—H38	119.9
H4B—C4—H4C	109.5	C40—C39—C44	119.0 (3)
N2—C5—C7	110.9 (2)	C40—C39—P2	121.6 (2)
N2—C5—C6	110.5 (3)	C44—C39—P2	118.9 (2)
C7—C5—C6	113.4 (3)	C39—C40—C41	120.0 (3)
N2—C5—H5	107.3	C39—C40—H40	120
C7—C5—H5	107.3	C41—C40—H40	120
C6—C5—H5	107.3	C42—C41—C40	120.8 (3)
C5—C6—H6A	109.5	C42—C41—H41	119.6
C5—C6—H6B	109.5	C40—C41—H41	119.6
H6A—C6—H6B	109.5	C41—C42—C43	119.4 (3)
C5—C6—H6C	109.5	C41—C42—H42	120.3
H6A—C6—H6C	109.5	C43—C42—H42	120.3
H6B—C6—H6C	109.5	C42—C43—C44	120.8 (3)
C5—C7—H7A	109.5	C42—C43—H43	119.6
C5—C7—H7B	109.5	C44—C43—H43	119.6
H7A—C7—H7B	109.5	C43—C44—C39	120.0 (3)
C5—C7—H7C	109.5	C43—C44—H44	120
H7A—C7—H7C	109.5	C39—C44—H44	120
H7B—C7—H7C	109.5	F1—C50—F3	108.2 (4)
C9—C8—C13	119.1 (3)	F1—C50—F2	106.8 (4)
C9—C8—C1	123.8 (2)	F3—C50—F2	106.0 (4)
C13—C8—C1	116.7 (2)	F1—C50—S1	112.2 (3)
C9—C8—Ru1	72.97 (16)	F3—C50—S1	112.2 (3)
C13—C8—Ru1	75.33 (16)	F2—C50—S1	111.1 (3)
C1—C8—Ru1	116.66 (18)	O6—C66—C68	120.7 (4)
C8—C9—C10	119.8 (3)	O6—C66—C67	121.9 (4)
C8—C9—Ru1	68.97 (15)	C68—C66—C67	117.4 (3)
C10—C9—Ru1	77.18 (16)	C66—C67—H67A	109.5
C8—C9—H9	120	C66—C67—H67B	109.5
C10—C9—H9	120	H67A—C67—H67B	109.5
Ru1—C9—H9	120	C66—C67—H67C	109.5
C11—C10—C9	121.1 (3)	H67A—C67—H67C	109.5
C11—C10—Ru1	75.44 (16)	H67B—C67—H67C	109.5
C9—C10—Ru1	66.20 (15)	C66—C68—H68A	109.5
C11—C10—H10	118.6	C66—C68—H68B	109.5
C9—C10—H10	118.6	H68A—C68—H68B	109.5
Ru1—C10—H10	118.6	C66—C68—H68C	109.5
O1—C11—C10	117.1 (3)	H68A—C68—H68C	109.5
O1—C11—C12	124.0 (3)	H68B—C68—H68C	109.5
C10—C11—C12	118.9 (3)	O5—C69—C70	122.4 (7)
O1—C11—Ru1	132.48 (19)	O5—C69—C71	117.0 (6)
C10—C11—Ru1	70.33 (16)	C70—C69—C71	120.6 (7)
C12—C11—Ru1	68.49 (15)	C69—C70—H70A	109.5
C13—C12—C11	120.4 (3)	C69—C70—H70B	109.5
C13—C12—Ru1	70.31 (15)	H70A—C70—H70B	109.5
C11—C12—Ru1	76.01 (16)	C69—C70—H70C	109.5
C13—C12—H12	119.6	H70A—C70—H70C	109.5
C11—C12—H12	119.6	H70B—C70—H70C	109.5
Ru1—C12—H12	119.6	C69—C71—H71A	109.5
C12—C13—C8	120.2 (3)	C69—C71—H71B	109.5
C12—C13—Ru1	74.01 (16)	H71A—C71—H71B	109.5
C8—C13—Ru1	66.65 (15)	C69—C71—H71C	109.5
C12—C13—H13	119	H71A—C71—H71C	109.5
C8—C13—H13	119	H71B—C71—H71C	109.5
Ru1—C13—H13	119	C1—N1—P1	116.5 (2)
C19—C14—C15	119.4 (3)	C1—N2—C2	121.7 (2)
C19—C14—P1	121.7 (2)	C1—N2—C5	121.8 (2)
C15—C14—P1	118.7 (2)	C2—N2—C5	116.5 (2)
C16—C15—C14	120.2 (3)	C11—O1—C26	118.4 (2)
C16—C15—H15	119.9	N1—P1—C14	102.38 (13)
C14—C15—H15	119.9	N1—P1—C20	102.62 (13)
C15—C16—C17	120.0 (3)	C14—P1—C20	98.99 (13)
C15—C16—H16	120	N1—P1—Ru1	106.16 (9)
C17—C16—H16	120	C14—P1—Ru1	127.40 (10)
C18—C17—C16	120.5 (3)	C20—P1—Ru1	116.18 (10)
C18—C17—H17	119.7	C39—P2—C27	99.05 (13)
C16—C17—H17	119.7	C39—P2—C33	106.32 (13)
C17—C18—C19	119.6 (3)	C27—P2—C33	103.16 (13)
C17—C18—H18	120.2	C39—P2—Ru1	123.58 (9)
C19—C18—H18	120.2	C27—P2—Ru1	115.43 (9)
C14—C19—C18	120.2 (3)	C33—P2—Ru1	107.27 (9)
C14—C19—H19	119.9	O3—S1—O2	114.85 (16)
C18—C19—H19	119.9	O3—S1—O4	114.49 (17)
C21—C20—C25	119.5 (3)	O2—S1—O4	114.44 (17)
C21—C20—P1	123.8 (2)	O3—S1—C50	103.96 (19)
C25—C20—P1	116.7 (2)	O2—S1—C50	103.62 (18)
C20—C21—C22	119.5 (3)	O4—S1—C50	103.45 (19)
C20—C21—H21	120.2	C8—Ru1—C9	38.06 (10)
C22—C21—H21	120.2	C8—Ru1—C13	38.02 (10)
C23—C22—C21	120.3 (3)	C9—Ru1—C13	67.02 (10)
C23—C22—H22	119.9	C8—Ru1—C12	66.99 (10)
C21—C22—H22	119.9	C9—Ru1—C12	77.96 (10)
C22—C23—C24	120.6 (3)	C13—Ru1—C12	35.68 (10)
C22—C23—H23	119.7	C8—Ru1—P1	77.72 (8)
C24—C23—H23	119.7	C9—Ru1—P1	106.01 (8)
C23—C24—C25	119.5 (3)	C13—Ru1—P1	83.61 (8)
C23—C24—H24	120.2	C12—Ru1—P1	113.63 (8)
C25—C24—H24	120.2	C8—Ru1—C10	66.47 (10)
C24—C25—C20	120.5 (3)	C9—Ru1—C10	36.61 (10)
C24—C25—H25	119.7	C13—Ru1—C10	75.79 (10)
C20—C25—H25	119.7	C12—Ru1—C10	63.45 (10)
O1—C26—H26A	109.5	P1—Ru1—C10	142.20 (7)
O1—C26—H26B	109.5	C8—Ru1—P2	120.95 (8)
H26A—C26—H26B	109.5	C9—Ru1—P2	93.03 (7)
O1—C26—H26C	109.5	C13—Ru1—P2	158.72 (7)
H26A—C26—H26C	109.5	C12—Ru1—P2	150.77 (8)
H26B—C26—H26C	109.5	P1—Ru1—P2	95.56 (3)
C28—C27—C32	118.6 (3)	C10—Ru1—P2	92.76 (7)
C28—C27—P2	123.2 (2)	C8—Ru1—C11	77.60 (10)
C32—C27—P2	118.1 (2)	C9—Ru1—C11	64.57 (10)
C29—C28—C27	120.6 (3)	C13—Ru1—C11	63.67 (10)
C29—C28—H28	119.7	C12—Ru1—C11	35.50 (10)
C27—C28—H28	119.7	P1—Ru1—C11	147.21 (7)
C30—C29—C28	120.1 (3)	C10—Ru1—C11	34.23 (10)
C30—C29—H29	120	P2—Ru1—C11	115.64 (7)
C28—C29—H29	120	C8—Ru1—Cl1	152.47 (8)
C29—C30—C31	119.8 (3)	C9—Ru1—Cl1	157.13 (8)
C29—C30—H30	120.1	C13—Ru1—Cl1	115.01 (7)
C31—C30—H30	120.1	C12—Ru1—Cl1	91.60 (7)
C32—C31—C30	120.5 (3)	P1—Ru1—Cl1	96.80 (3)
C32—C31—H31	119.7	C10—Ru1—Cl1	120.54 (7)
C30—C31—H31	119.7	P2—Ru1—Cl1	86.24 (3)
C31—C32—C27	120.4 (3)	C11—Ru1—Cl1	95.17 (7)
